# Dr. David Everett Wheeler

**Published:** 1918-10

**Authors:** 


**Dr. David Everett Wheeler** of Buffalo was killed Aug. 13 while attending the wounded under fire. He graduated at the College of Physicians and Surgeons of N. Y. (Columbia) in 1898 and was about 46 years old. He went to Europe as a Red Cross worker in the first year of the war, enlisted in the Foreign Legion of France Feb. 15, 1917 and was wounded in the leg in the Champagne battle, Sept. 28. While crawling to the rear, he stopped to minister to others and was awarded the Croix de Guerre for heroism. Later he entered the Canadian Army as Captain and was transferred to the U. S. Army on our entrance into the war. He was later made a regimental surgeon with rank of Major. Before the war, his adventurous spirit led him to make several hunting trips in the far north.

